# Supplementary material for: Characterization of a set of abdominal neuroendocrine cells that regulate stress physiology using colocalized diuretic peptides in Drosophila
Source: Cell Mol Life Sci. 2017 Oct 17;75(6):1099–115. doi: 10.1007/s00018-017-2682-y (PMC5814475; doi:10.1007/s00018-017-2682-y)
Supplement: Supplementary file 1 — Supplementary material 1 (PDF 3096 kb) [file 18_2017_2682_MOESM1_ESM.pdf]

Supplementary material

Characterization of a set of abdominal neuroendocrine cells that regulate stress physiology using colocalized diuretic peptides in *Drosophila*

Meet Zandawala[1], Richard Marley[2], Shireen A. Davies[2] and Dick R. Nässel[1]

1. Stockholm University, Department of Zoology, Stockholm, Sweden
2. Institute of Molecular, Cell and Systems Biology, College of Medical, Veterinary and Life Sciences, University of Glasgow, Glasgow, UK

E-mail: dnassel@zoologi.su.se and meet.zandawala@zoologi.su.se

Supplementary Figures 1 – 7 (Fig. S1- Fig. S7)

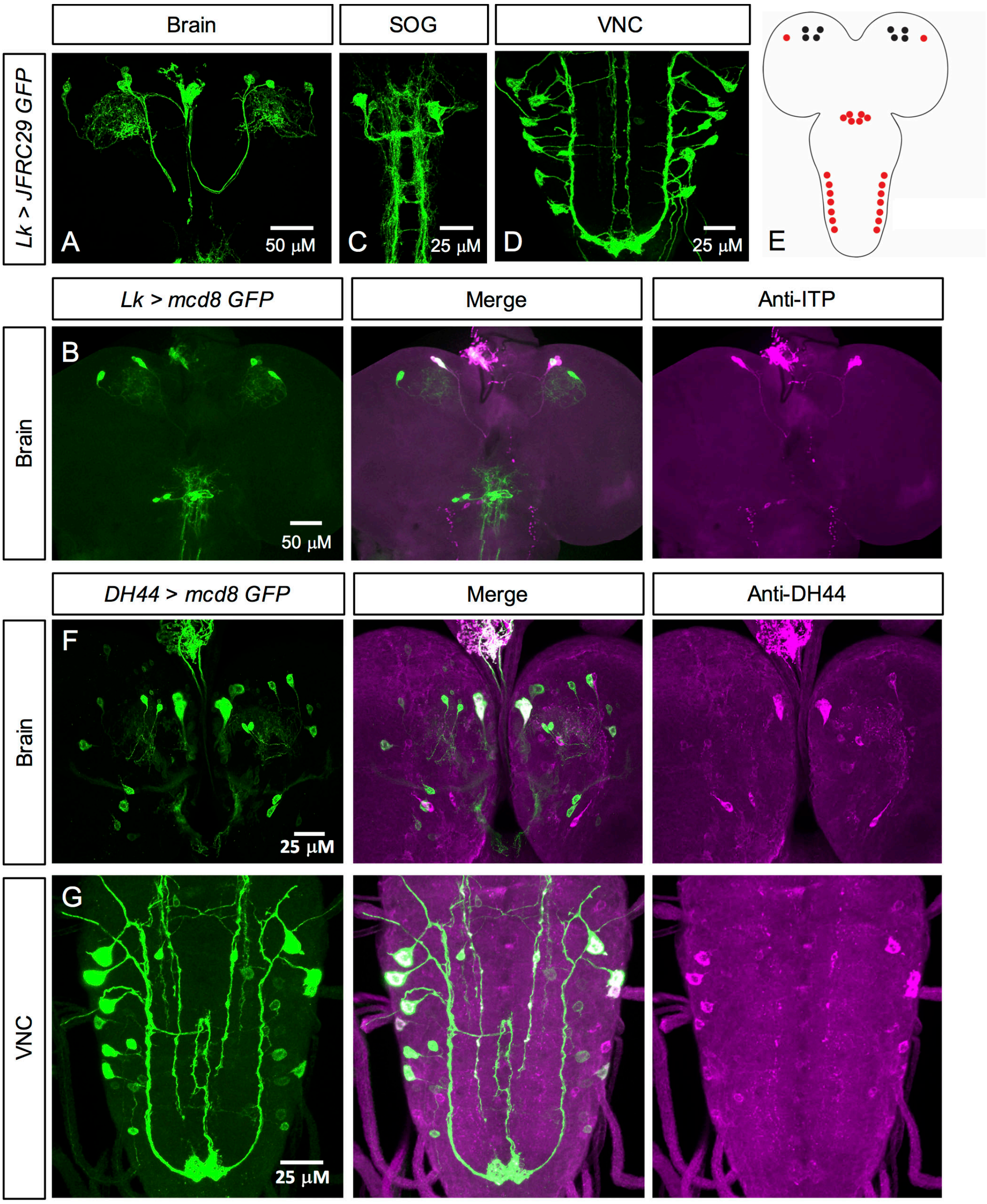

Figure S1: LK and DH44 expression in the larval *Drosophila* CNS. (A) *Lk-GAL4* drives expression in five pairs of neurons in the brain; however, four of these pairs do not display any LK-immunoreactivity [20]. **(B)** These four pairs of neurons display ITP-immunoreactivity. **(C)** Three pairs of neurons in the subesophageal ganglion express *Lk* in larval *Drosophila*. **(D)** Seven pairs of neurons in the larval ventral nerve cord (VNC) express *Lk*. **(E)** A schematic of LK-expressing neurons in the larval brain and VNC of *Drosophila.* Neurons displaying LK-immunoreactivity are labelled in red and neurons displaying ITP-immunoreactivity are labelled in black. **(F)** *DH44-GAL4* driven GFP and DH44-immunoreactivity is present in three pairs of median neurosecretory cells in the larval brain. **(G)** DH44 is expressed in several neurons, with strong expression seen in seven pairs of neurosecretory cells in the larval VNC. In both F and G, there are some neurons that contain GFP but do not contain DH44-immunoreactivity.

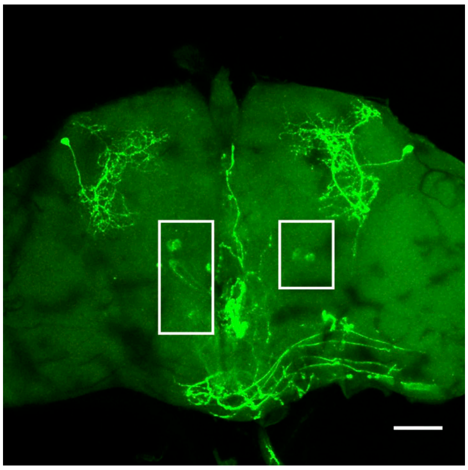

Figure S2: LK expression in adult *Drosophila* brain. LK-*GAL4* drives weak GFP expression in four pairs on neurons in the adult brain. The location of these cells is indicated by white boxes. Scale bar: 50 µm.

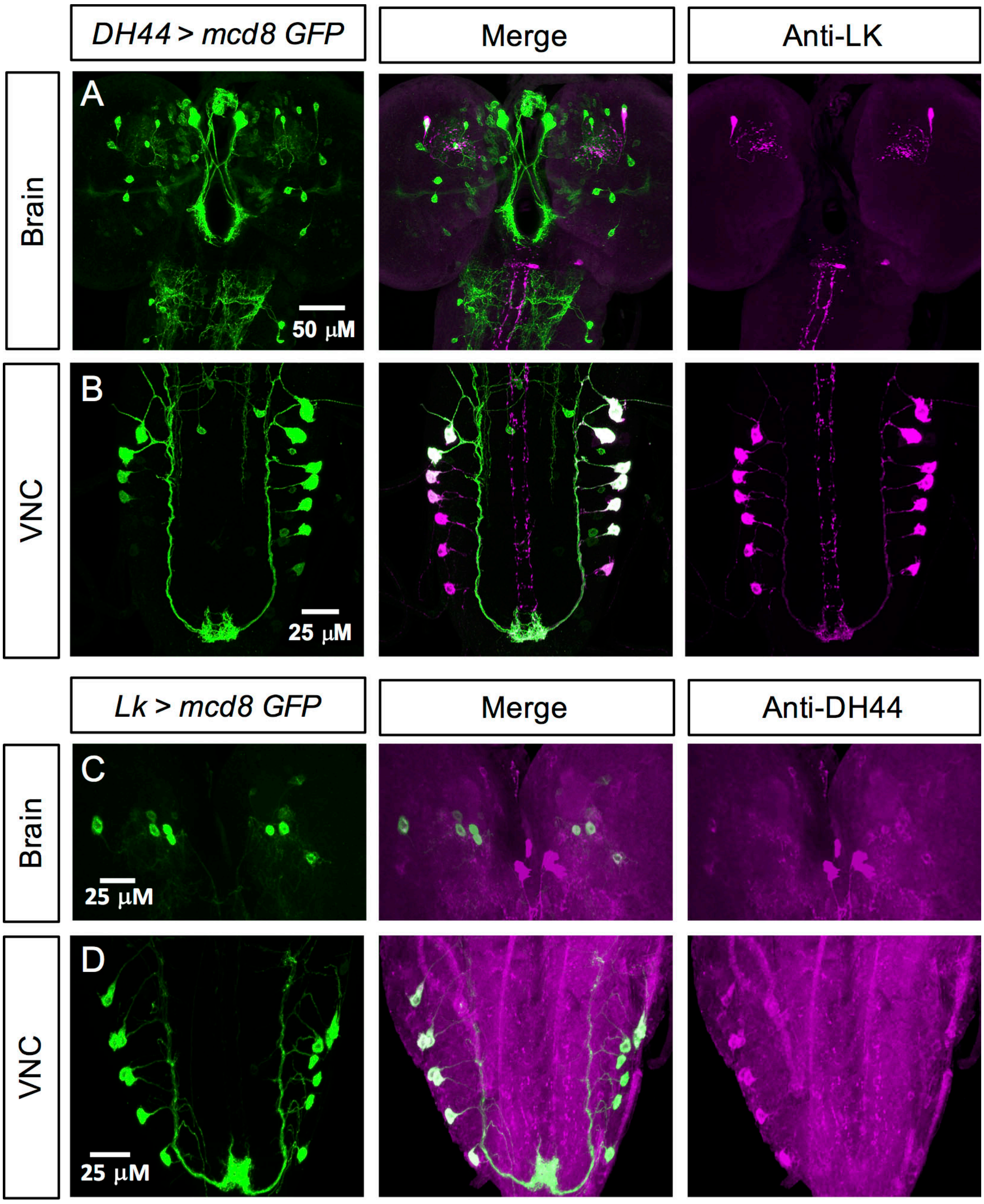

Figure S3: LK and DH44 are co-expressed in neurons of the ventral nerve cord but not in the brain of larval *Drosophila*. (A) *DH44-GAL4* driven GFP is not co-localized with LK-immunoreactivity in the larval brain. **(B)** *DH44-GAL4* driven GFP is co-localized with LK-immunoreactivity in all seven pairs of abdominal LK neurons (ABLKs) in the ventral nerve cord (VNC) **(C)** *Lk-GAL4* driven GFP is not co-localized with DH44-immunoreactivity in the larval brain. **(D)** *Lk-GAL4* driven GFP is co-localized with DH44-immunoreactivity in ABLKs in the larval VNC.

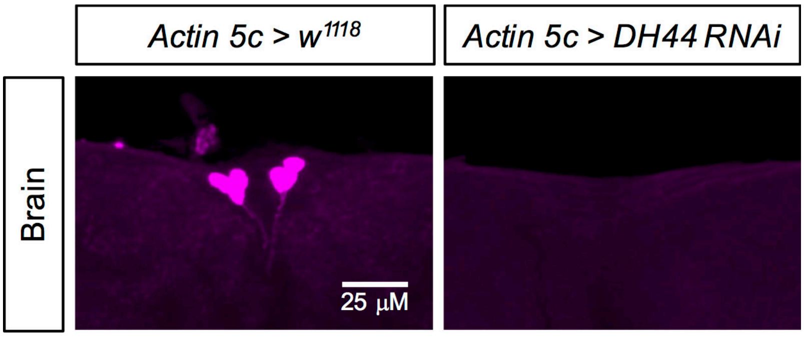

Figure S4: Knockdown of *DH44* using *Actin5c-GAL4*. *Actin5C-GAL4* driven *DH44* knockdown results in a complete abolishment of DH44-immunoreactivity in the six neurons in pars intercerebralis.

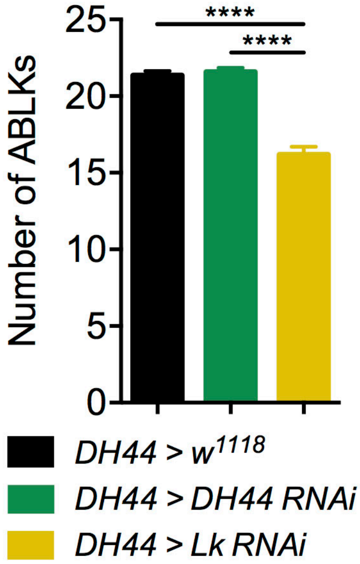

Figure S5: Number of LK-immunoreactive neurons in the VNC following knockdown of *DH44* and *Lk* using *DH44-GAL4*. *Lk* knockdown but not *DH44* knockdown causes a significant decrease in the number of LK-immunoreactive neurons that could be detected in the adult VNC. (**** $p < 0.0001$, as assessed by One-way ANOVA).

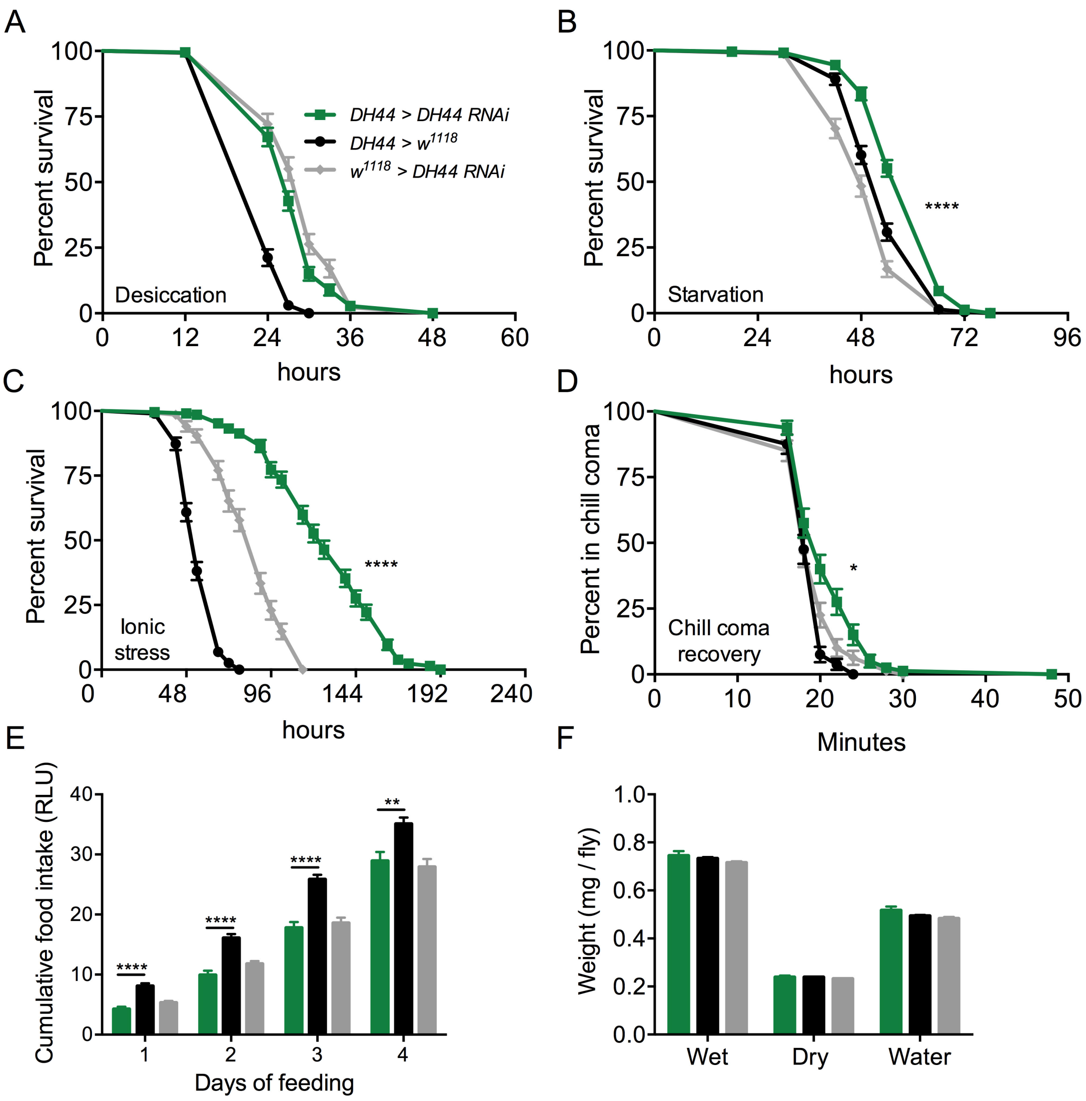

Figure S6: Knockdown of *DH44* using *DH44-GAL4* impacts stress resistance and feeding in *Drosophila*. *DH44-GAL4* driven *DH44* knock down results in a significant increase in survival compared to control flies under **(A)** desiccation (compared to the GAL4 control), **(B)** starvation and **(C)** ionic stress (artificial food supplemented with 4% NaCl). Data are presented in survival curves and the error bars represent standard error (**** $p < 0.0001$, as assessed by Log-rank (Mantel-Cox) test) **(D)** *Lk* knock down results in a delayed recovery from chill coma. (* $p < 0.05$, as assessed by Log-rank (Mantel-Cox) test) **(E)** There is no significant difference (One-way ANOVA) in feeding as measured by capillary feeding (CAFE) assay between *Lk* knock down and control flies. Results are presented as cumulative food intake over four days. **(F)** There is no significant difference in wet weight, dry weight and water content of *DH44*-knockdown and control flies. Legend for B-F is the same as the one in A.

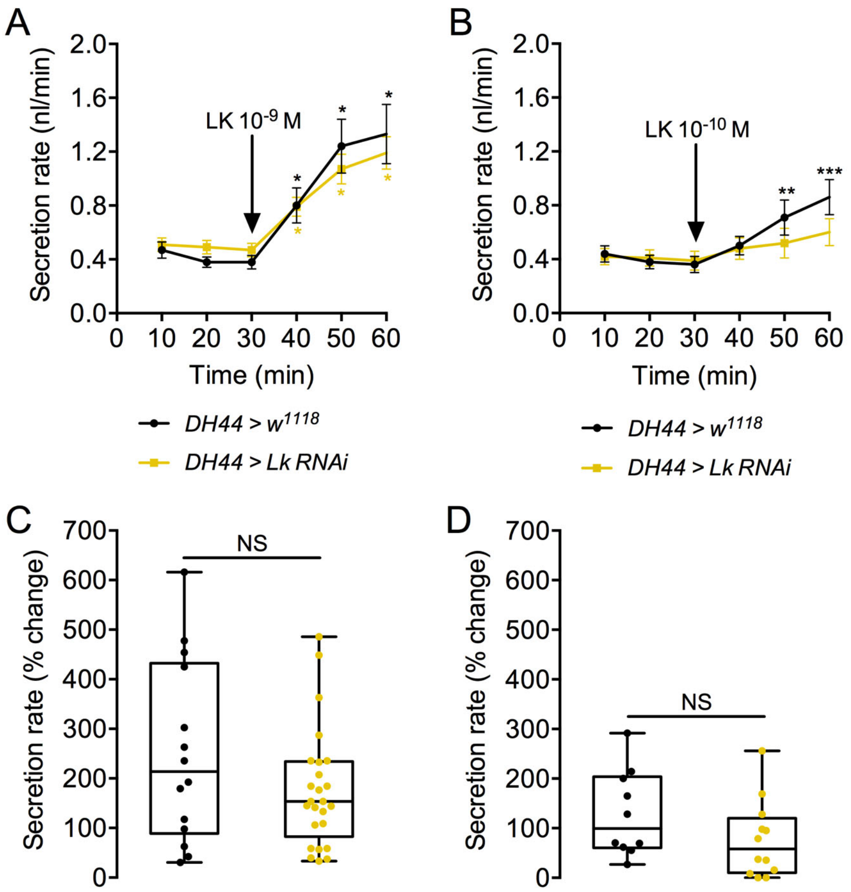

Figure S7: Knockdown of *Lk* in ABLKs with *DH44-GAL4* does not influence LK-stimulated Malpighian tubule secretion *ex vivo*. (A) Secretion rates of 10^{-9} M LK stimulated MTs isolated from *DH44* > w^{1118} (*n* = 14) or *DH44* > *Lk RNAi* flies (*n* = 25). **(B)** Secretion rates of 10^{-10} M LK stimulated MTs isolated from *DH44* > w^{1118} (*n* = 10) or *DH44* > *Lk RNAi* flies (*n* = 12). For both A and B, secretion rates were measured at 10 min intervals for 30 min before and after the addition of peptide (indicated with an arrow). **(C, D)** Change (%) in secretion determined by comparing the secretion rate over the first 30 min to the maximum secretion rate following peptide application. The legend and sample size for C and D are the same as the one in A and B, respectively. Asterisk indicates significantly different secretion rate compared to basal secretion rate (secretion rate prior to the addition of peptide). (NS = not significant, * $p < 0.05$, ** $p < 0.01$, *** $p < 0.001$; Mann-Whitney U test).
